# IV-Range Carrot Waste Flour Enhances Nutritional and Functional Properties of Rice-Based Gluten-Free Muffins †

**DOI:** 10.3390/foods13091312

**Published:** 2024-04-24

**Authors:** Claudia Bas-Bellver, Cristina Barrera, Noelia Betoret, Lucía Seguí, Joanna Harasym

**Affiliations:** 1 Instituto Universitario de Ingeniería de Alimentos—FoodUPV, Universitat Politècnica de València, Camino de Vera s/n, 46022 Valencia, Spain; clbabel@etsiamn.upv.es (C.B.-B.); mcbarpu@tal.upv.es (C.B.); noebeval@upvnet.upv.es (N.B.); 2 Department of Biotechnology and Food Analysis, Wrocław University of Economics and Business, Komandorska 118/120, 53-345 Wrocław, Poland; joanna.harasym@ue.wroc.pl

**Keywords:** food waste valorization, gluten-free bakery products, vegetable flours, carrot residues, sustainable diets, nutritional foods, functional ingredients

## Abstract

Fortification of bakery products with plant-based functional ingredients has gained interest in recent years. Low-cost fruit and vegetable waste has been proposed to replace wheat flour, but less research has been conducted on gluten-free flours. Rice is generally accepted as a gluten-free alternative to wheat flour but is poor in bioactive constituents; thus, the addition of vegetable-based functional ingredients could improve the nutritive value of gluten-free products. In the present work, IV-range carrot waste powder (CP) was incorporated into rice-based gluten-free muffin formulations in different proportions (5, 10, 20, and 30% *w*/*w*). The impact of CP addition on physicochemical and antioxidant properties was evaluated in flour blends, doughs, and baked products. Products were also evaluated in terms of water activity, hardness, and colour before and after a one-week storage period under fridge conditions. The results showed that water and oil absorption capacities increased in flour blends with CP addition, whereas the pasting properties of flour blends were affected when adding CP. Rheological measurements revealed an increase of G’ and G’’ modulus values with CP addition. Colour was also significantly modified by CP addition, since CP provided an orangish and brownish colour, but also due to intensified Maillard reactions during baking. Muffin hardness was reduced in enriched formulations compared to control ones, which was attributed to the fibre being incorporated with CP. It was confirmed that CP addition improved the antioxidant properties of both flour blends and muffins, with the higher the replacement, the better the antioxidant properties. The quality of gluten-free muffins was hindered after one week stored under cold conditions, so that colour was affected, hardness increased, and the antioxidant properties diminished. In conclusion, this work presents an interesting approach for the use of carrot waste flour as a functional food ingredient to improve the nutritional value of new gluten-free rice-based muffins, thus contributing to the circularity of food systems and to the development of healthier and more sustainable diets.

## 1. Introduction

Development of new food products must consider both more nutritious and more sustainable approaches, which converge in the concept of sustainable healthy diets [1]. From the nutritional point of view, new food development focuses on foods with fewer calories, more dietary fibre, and less salt and additives, resulting in improved diet quality and better human health [2]. Obesity and non-communicable chronic diseases related to poor dietary habits are public health issues that need to be addressed, so that consumption patterns ought to shift toward diets [3] including more fruits and vegetables, whole grain products, legumes, and plant-based oil sources. On the other hand, the sustainability and circularity approach for new food designs aims to minimize food waste and, if inevitably produced, it encourages its valorization and reintroduction into the food chain. According to the FAO, one third of the food produced is lost or wasted, so reducing this wastage is one of the aims of the 2030 Agenda for Sustainable Development [4]. Contrarily, consumer trends based on convenient on-the-go foods, such as IV-range products, generally imply increased food waste generation at the production level. Nevertheless, even if this could be considered a negative point, generating these residues at the industry rather than household level concentrates their production and provides an opportunity to valorize them. This was the basis of previous research approaches [5,6] in which upcycled powdered ingredients were obtained from vegetable waste generated at the early stages of processing.

Fruit and vegetable by-products are a rich source of dietary fibre, minerals, vitamins, antioxidants, and bioactive compounds, which might have a positive impact on human health [7,8]. In particular, carrot (*Daucus carota* L.) residues are a well-known source of phenolics, flavonoids, ascorbic acid, and carotenoids [9]. Carotenoids are natural colorants with important physiological activities that benefit human health by enhancing the immune system, protecting against solar radiation, maintaining the visual system, and reducing the risk of cardiovascular disease and certain types of cancer [10,11,12,13]. Fruit and vegetable residues are highly perishable goods, but they can be transformed into dried powdered products with preserved bioactive compounds, thus extending their shelf life and improving their versatility, as demonstrated previously [5,6]. One wise strategy for the valorization of these upcycled ingredients is to incorporate them into different food matrices as functional food ingredients [14].

Bakery products offer attractive characteristics to consumers, such as a relatively long shelf life, high convenience, and organoleptic quality [15]. Particularly, muffins are popular, widely consumed wheat-based products [16]. However, gluten present in wheat may originate adverse reactions in individuals with celiac disease and those who are allergic or intolerant to gluten proteins, for whom the only effective treatment is adhering to a gluten-free diet [16,17]. Due to the increasing prevalence of gluten-related conditions, the global gluten-free bakery market is witnessing a significant surge, which has boosted the demand for gluten-free baked goods. In addition, increasingly healthy individuals are reducing gluten consumption because of a “health halo” effect [18]. However, many commercial gluten-free products are characterized by unsatisfactory texture, low nutritional quality, and short shelf life [18]. In fact, developing new gluten-free bakery products with nutritional and organoleptic properties similar to their gluten-based counterparts is rather challenging [19]. As a wheat flour substitute, rice flour is considered a good cereal source for obtaining gluten-free foodstuffs, though nutritionally poor after bran removal. In this context, fortification of gluten-free flours and products with plant-based ingredients is an interesting approach that needs to be better explored.

Fortification of bakery products with plant-based functional ingredients has gained interest in recent years [20]. Low-cost fruit and vegetable waste has been proposed to replace wheat flour for the production of cake, bread, muffins, or breadsticks using potato, mango, kinnow, pomegranate, and grape pomace or peel powder [21,22,23,24,25] or [14]. However, less research has been conducted on gluten-free flours [26]. Application of fruit and vegetable discards in gluten-free bakery products is relatively new [23]. Some examples include muffins enriched with mushroom or carrot pomace powder [16] or cakes fortified with apple, orange, or carrot pomace powder. Among carrot by-products, carrot pomace has already been proposed as a functional ingredient [9,17], but to the best of our knowledge, IV-range carrot waste has not been explored to date.

In the present work, carrot waste powder (CP) obtained from a IV-range carrot production line as explained elsewhere [27,28] was incorporated into rice-based gluten-free muffins formulations in different proportions to evaluate its impact on the physicochemical and antioxidant properties of the blends, doughs, and products obtained. Hence, upcycling carrot waste into functional food ingredients to fortify rice-based muffin formulations is examined as a potential alternative to produce healthier and more sustainable value-added baked goods.

## 2. Materials and Methods

### 2.1. Raw Materials and Ingredients

Carrot (*Daucus carota*) waste, consisting of discarded sticks of a IV-range carrot processing line, was transformed into carrot waste powder (CP), as explained elsewhere [27,28]. For the present application, powder hot-air dried at 70 °C was selected, so that carrot waste was transformed by first chopping with a blade mill to obtain pieces of ≤10 mm diameter (Thermomix^®^ TM6, Vorwerk, Madrid, Spain), then hot-air drying at 70 °C in a pilot plant convective tray dryer with transversal flux (Pol-eko Aparatura, Katowice, Poland) until reaching a water activity value below 0.3, and finally, milling in a food processor (Thermomix^®^ TM6, Vorwerk, Madrid, Spain) at 10,000 rpm for 2 min at 30 s intervals to obtain a fine powder with an equivalent volume diameter (D [3,4]) of about 200 μm [27,28]. Other ingredients used in the formulations, i.e., rice flour, eggs, sugar, margarine, and baking powder, were purchased in a local supermarket in Wroclaw (Poland).

### 2.2. Muffin Preparation

Muffins were prepared following the protocol of Olawuyi and Lee [16]. The muffin dough was formulated using 200 g of flour, 4.8 g of baking powder, 150 g of margarine, 150 g of sugar, and 187 g of whisked eggs. Rice flour (RF) was partially replaced with carrot powder (CP) at different mass percentages (*w*/*w*): 5% (CP 5%), 10% (CP 10%), 20% (CP 20%), and 30% (CP 30%), which were established based on previous experience and preliminary testing, while control muffins were prepared with RF exclusively. After homogenously mixing all of the ingredients, around 100 g of dough was scooped in muffin paper cups and baked in an oven (MPM model MPE-08/T, Milanówek, Poland) at 180 °C for 30 min. Six muffins were obtained per formulation, in two baking batches (three replicates each).

### 2.3. Characterization of Rice Flour and Flour Blends

#### 2.3.1. Physicochemical Properties

The moisture content of the flour blends was obtained according to Official Method AACC44-19 (AACC, 2000) by drying in an air oven at 135 °C until constant weight. The water absorption capacity (WAC) and oil absorption capacity (OAC) of the flour mixtures were determined by the centrifugation method described by Harasym et al. [29] as follows: 1 g of sample was mixed with 10 mL of distilled water or corn oil, respectively. Dispersions were vortexed during 30 s at high speed (Heidolph Reax, Schwabach, Germany) and then centrifuged for 25 min at 3000× *g* (Thermo Fisher Scientific, Waltham, MA, USA). After centrifugation, the supernatant was removed and the precipitate was weighed. The results were expressed as g of water or oil retained per g of flour. The hydrophilic/lipophylic index (HLI) was calculated as the ratio between WAC and OAC.

#### 2.3.2. Pasting Properties of Flour Blends

The viscometric profile of the flour blends was obtained by following ICC Standard method 162 with a Rapid Visco Analyser (RVA-4500, Perkin Elmer, Waltham, MA, USA) according to the methodology described by Harasym et al. [29]. Thus, 2.5 g of sample was transferred to an RVA container with the amount of distilled water (as solvent) adjusted to the total weight of 28.5 g. Each flour suspension was equilibrated at 50 °C for 1 min, then the temperature was raised to 95 °C at a rate of 5 °C/min, held at 95 °C for 5 min, then cooled to 50 °C at a rate of 5 °C/min, and finally held at 50 °C for 4 min. The stirring speed was set at 960 rpm for the first 10 s and then maintained at 160 rpm for the rest of the analysis. The results of peak viscosity (PV), trough viscosity (TV), breakdown (BD = PV − TV), final viscosity (FV), setback (ST = FV − TV), pasting temperature, and peak time parameters were obtained.

### 2.4. Characterization of Muffin Batters and Products

#### 2.4.1. Rheological Measurements

Dynamic oscillatory tests of the muffin batters were performed with an Anton Paar MC102 rheometer (Anton Paar., Stuttgart, Germany) using a parallel plate geometry (40 mm diameter) of serrated steel surfaces with 1 mm of working gap and with the temperature set at 25 °C, controlled by a KNX2002 thermal controller. Viscoelastic behaviour was expressed in terms of the storage or elastic modulus (G’) and the loss or viscous modulus (G’’) with a frequency sweep performed from 10 to 1 Hz in the linear viscoelastic region at a constant stress of 1 Pa.

#### 2.4.2. Physicochemical Characterization of Muffins

The water activity (a_w_) of the baked muffins was determined with an AquaLab 3TE analyzer (Decagon Devices, Inc., Pullman, WA, USA). Weight was measured using an Axis AD1000 electronic balance (Axis, Gdansk, Poland). Volume was measured using a 3D Scanner v2 with Quickscan (Matter and Form Inc, Toronto, Ontario, Canada) analyser. After careful separation with a knife, the colour of the crust and the crumb of the muffins was measured with a Konica Minolta CR-310 chroma meter (Konica Minolta, Ramsey, NJ, USA) using a D65 standard illuminator and the 2° standard observer. CIEL*a*b* coordinates were obtained as colour parameters, and the total colour change after one week of storage (∆E) was calculated according Equation (1):

(1) $$\Delta E=\sqrt{{\left(L_{i}^{*}-L_{n}^{*}\right)}^{2}+{\left(a_{i}^{*}-a_{n}^{*}\right)}^{2}+{\left(b_{i}^{*}-b_{n}^{*}\right)}^{2}}$$

where L_i_*, a_i_*, and b_i_* are the colour parameters of freshly prepared muffins and L_n_*, a_n_*, and b_n_* are those of muffins stored for one week.

The texture of the muffins was measured with an AXIS texture analyser (Axis, Gdansk, Poland) using the software “FM AXIS” and an aluminium 20 mm diameter cylindrical probe. A compression test was performed on 2 cm-thick crumb slices, which were subjected to 50% deformation at 1 mm/s speed test. Compression maximum hardness values (N) were registered.

### 2.5. Antioxidant Activity, Total Phenolic and Reducing Sugar Contents

The antioxidant properties were measured in the flour blends and muffins (both crust and crumb). For antioxidant compound extraction, 0.5 g of sample was mixed with 8 mL of water and agitated in a laboratory orbital shaker (WU4, Premed, Marki, Poland) at room temperature for 2 h. The mixture was then centrifuged at 3500 × *g* for 10 min (MPW-350, MPW, Warszawa, Poland) and the supernatant collected. The extracts obtained were then used for antioxidant determinations.

Total phenolic content was determined using the Folin-Ciocalteu method [29]. A 20 μL volume of the extract was mixed with 1.58 mL of distilled water and 100 μL of Folin-Ciocalteu reagent. After 5–8 min of incubation at room temperature, 300 μL of a saturated solution of Na_2_CO_3_ was added. The mixture was incubated at 38 °C for 30 min (MLL147, AJLElectronics, Kraków, Poland), and after that, the absorbance was measured at 765 nm (UV-VisUltrospec 2000, Pharmacia Biotech, Piscataway, NJ, USA). The results were expressed as mg of gallic acid equivalent (GAE) per g of sample.

Reducing sugar content was measured using the DNS (dinitrosalicylic acid) method with some modifications [30,31]. A 0.5 mL volume of the extract was mixed with 0.25 mL of DNS reagent (1% of 3,5-dinitrosalicylic acid solution in 0.4 M NaOH). The mixture was incubated in a boiling water bath for 5 min, followed by cooling to 50–60 °C. After the addition of 3 mL of distilled water, the absorbance was measured at 530 nm. The results were given as mg of glucose equivalent (GE) per g of sample.

The DPPH method [32] was carried out to determine the antioxidant capacity of the samples. A 34.5 μL volume of the extract was mixed with 1000 μL of 0.1 mM of DPPH solution (2,2-diphenyl-1-picrylhydrazyl) in methanol with an absorbance of 0.9 ± 0.1 measured at 517 nm. After 20 min of incubation at room temperature, absorbance was measured at 517 nm. The antioxidant capacity was expressed as μmol of trolox equivalent (TE) per g of sample.

### 2.6. Evolution of Muffin Properties during Cold Storage

The stability of muffin properties after a short-term cold storage period was also investigated. Therefore, muffin properties were evaluated 1 h after production and after 7 days of storage in hermetic bags at 4 °C to evaluate changes in water activity, colour, texture (hardness), and antioxidant properties. The plastic bag material consisted of 2 layers: a 60 μm inner layer of food contact polyethylene and an outer layer of 15 μm polyamide (nylon), which increased strength and guaranteed airtightness. These materials acted as a UV filter.

### 2.7. Statistical Analyses

All measurements were made at least in triplicate. The analysis of variance (ANOVA) of the results was evaluated with Statgraphics Centurion software (Centurion XVII.I version, StatPoint Technologies, Inc., Warrenton, VA, USA). ANOVA was performed with a previous normality of checked data using a *p*-value < 0.05 significance level.

## 3. Results and Discussion

### 3.1. Characterization of Flour Blends

The moisture content, water absorption capacity (WAC), oil absorption capacity (OAC), and hydrophilic/lipophilic index (HLI) of the different flour blends are presented in Table 1. Significant differences were found between rice flour and CP-enriched flour moisture contents, with rice flour showing the highest value, since carrot powder initially presented with lower moisture content, about 2–3% [6,27], which resulted in lower values when mixed with rice flour.

The analysis of water and oil interaction properties (WAC and OAC) of the flour blends revealed that the addition of CP to rice flour induced a statistically significant increase in both parameters, and the same occurred with HLI values. Similar results have previously been reported [28,33] when adding vegetables to rice or wheat flour, the latter replaced with carrot pomace. This increase has been attributed to the higher fibre content of flours enriched with vegetable waste powders, since fruit and vegetable by-products are characterized by their high fibre content [34]. Fibre competes with rice flour for water and oil absorption so that the WAC and OAC values in enriched flours were significantly increased. Among enriched flours, WAC values varied from 2.43 ± 0.05 to 3.56 ± 0.12 mL/g, with the flour with 30% of CP exhibiting the highest value. OAC values did not show significant differences between enriched flours. Carrot waste flour blends showed better water absorption than oil absorption capacity, suggesting a major hydrophilic character, as previously evidenced for carrot powders [5].

**Figure 1:**
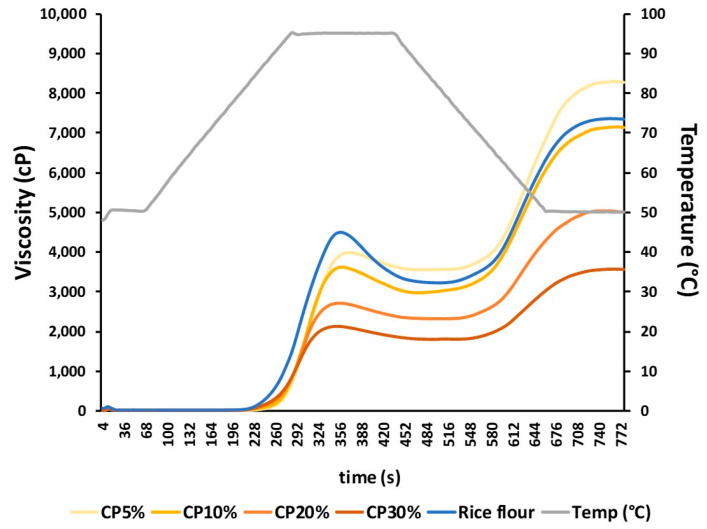
Pasting curves of rice flour (RF) and flours enriched with carrot powder (CP). 5, 10, 20, 30% indicate the percentage of residue powder in the flour blend.

The pasting properties of rice flour and CP flour blends are summarized in Table 2, while the pasting curves are presented in Figure 1. As observed, the incorporation of CP significantly modified the pasting properties and decreased the viscometric parameters (peak, trough, setback, and final viscosities) compared to rice flour (Table 2, Figure 1), thus indicating greater structural rigidity when CP was added. The modification of dough behaviour could be partially explained by the increased water absorption (Table 1) and binding capacity as a consequence of the higher fibre content of the enriched doughs [24] due to carrot powder addition (11.71 ± 0.12% [16]). These results aligned with previous studies in which other vegetable-enriched flour blends have been evaluated, namely mushroom and carrot pomace, kinnow peel, white cabbage waste, or chestnut flour [24,28,35]. As in the present study, the results evidenced that vegetable powders induced a decrease in the pasting parameters of starchy flours, which was attributed to the higher water-holding and binding ability promoted by fibre addition [24]. In the present work, the pasting temperature slightly decreased with CP addition, which indicated a fall in the gelatinization temperature [35]. Rice flour, with the highest pasting temperature, was more resistant to swelling and rupturing due to a larger starch content [33], although no statistically significant differences were observed in the peak temperature parameter among flour blends. However, besides fibre competition for water, it has also been reported that phenolic compounds may also modify the properties of starch-based doughs due to interactions between starch and phenolic constituents [36]. This effect appears to be dependent on the type of starch, chemical composition of the extract, the structure of the phenolic compound, and the pH of the system. An example would be that catechin or gallic acid addition decreased the hot paste viscosity of rice starch and increased that of potato starch, or that black tea extract was more effective in reducing the cold paste viscosity of wheat, maize, potato, and rice starches than green tea extract. As for pH, the addition of phenolics or extracts to doughs may modify pH and, therefore, the pasting properties.

Among the properties of flour blends, their pasting properties play a crucial role in the range of uses and potential applications. The pasting characteristics of flours are primarily reliant on the rigidity of the starch granule, which in turn affects the granule swelling potential and the amount of amylose leaching out into the solution [37]. The observed negative effect of CP addition on the viscoelastic properties of the doughs could have negatively impacted the baked products obtained, as reduced pasting properties imply a diminished ability of the dough to deform and retain gas bubbles, thus limiting the application of flour blends. This limitation could be more significant in products with a more sponge-like structure, such as muffins, cakes, or bread.

The antioxidant properties of rice flour and the different flour blends are listed in Table 3. The antioxidant potential of IV-range carrot waste powder has been evidenced in previous studies [5,6,27], with values ranging from 2 to 4.5 mg GAE per gram of dry matter and from 1.8 to 4.8 µmol of TE per gram of dry matter, respectively. Other bioactive compounds, such as carotenoids, have also been identified in powdered carrot waste. Among carotenoids, CP was particularly rich in α- and β-carotene, with a total carotenoid content ranging from 56 to 68 μg/g_dm_ when dried at 70 °C [27]. As expected, blending rice flour with CP had a positive impact on the antioxidant properties of the flour blends. The reducing sugar content, total phenol content, and antioxidant activity determined by the DPPH method exhibited progressive increases in the flour blends with the percentage of replacement as a result of the incorporation of the antioxidant compounds contained in the carrot waste powder. Other researchers have also reported an improvement in the antioxidant properties of flour blends by adding carrot and mushroom [16] or white cabbage powder [28] to rice flour. These results demonstrated the enhanced nutritional attributes of gluten-free mixtures enriched with CP, which could result in higher nutritional quality of baked muffins.

### 3.2. Muffin Dough and Product Characterization

**Figure 2:**
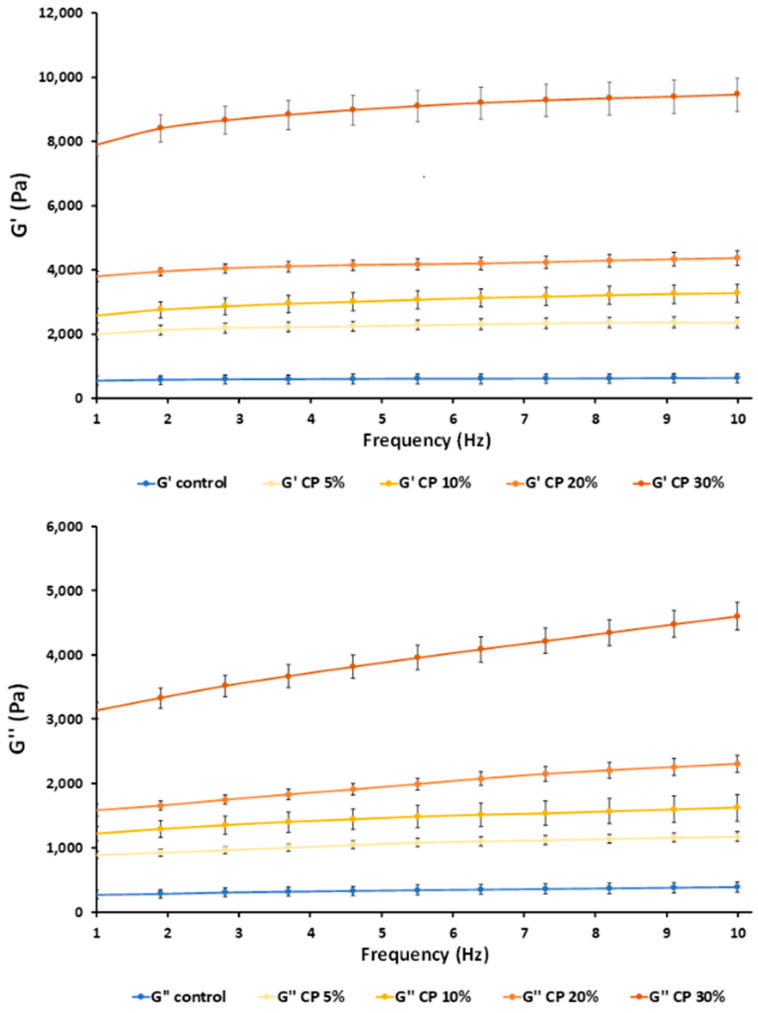
Rheograms of gluten-free muffin batters, obtained by partially replacing rice flour with carrot waste powder (CP). G’: storage modulus, G’’: loss modulus. 5, 10, 20 and 30% indicate the percentage of carrot waste powder in the flour blend.

The viscoelastic behaviours of the different dough formulations are represented in Figure 2. The storage or elastic (G’) and loss or viscous (G″) moduli slightly increased with an increase in frequency, both showing a frequency dependence. As observed, G’ was higher than G’’ in the whole frequency range in all cases, indicating the elastic behaviour of batters. Likewise, the loss tangent (tan δ = G″/G’) values were in all cases in the range of 0.4–0.6 (tan δ < 1) and slightly dependent on frequency, which explained the rheograms of gels with more solid-like behaviour [33]. This behaviour has also been observed in other enriched rice-based batters [17,28,38].

The incorporation of CP significantly increased the G’ and G″ values compared to the control, indicating higher viscoelastic behaviour. This increase was observed to be proportional to the amount of CP added. This increase in storage (G′) and loss (G″) modulus values was justified by the strengthening action of added functional ingredients due to crosslinking of the polymer system and the formation of large insoluble polymers [39].

Table 4 shows the water activity (a_w_), weight, and volume of the muffins, and a_w_ after one week of cold storage. Muffins formulated with rice and CP presented lower a_w_ values than control muffins, both before and after storage. This result was consistent with the higher moisture content of rice flour compared to powder-enriched ones (Table 1), even if no significant differences were found among CP-enriched flours. A slight a_w_ increase was observed after storage, but it was not very significant. In enriched formulations, weight and volume usually decreased when increasing the percentage of replacement: M5 having the highest values and M30 having the lowest ones. As reported by Olawuyi and Lee [16], the addition of mushroom and carrot powders to muffin formulations increased their weights due to fibre addition. Another study about refined wheat flour mixed with kinnow peel powder suggested that higher fibre content results in less moisture loss during baking, thus increasing muffin weight [23]. Contrarily, and in line with the results of the present paper, other researchers have reported a volume decrease when incorporating pomace powder into cakes [17] and muffins [16] and have attributed this result to the increased fibre content, which worsens the viscoelastic behavior of doughs. However, as previously discussed, phenol–starch interactions may also negatively affect the pasting properties and be responsible for this volume reduction [36]. Hence, reduced viscoelastic properties due to fibre competition for water and/or phenolic compounds incorporated with CP might have led to reduced leavening ability, fewer trapped air bubbles, and increased compactness.

The colour parameters (L*, a*, b*, and ΔE) of the muffins (crust and crumb) are presented in Table 5. Statistically significant differences were obtained among formulations. The crust and crumb lightness (L*) of rice muffins (control) were significantly higher than in CP enriched muffins, whereas the L* values of the muffin crumb and crust decreased with CP addition. The natural brownish colour of CP decreased the brightness of muffins when incorporated into formulations. In addition, reducing sugars present in carrot powder and blends could have contributed to non-enzymatic Maillard reaction during baking, thus reducing lightness. Similar results were obtained by Olawuyi and Lee [16] and Kirbas et al. [17] in gluten-free carrot powder-based muffins and cakes, respectively.

The a* values for the crust and crumb increased with the increase in CP. The darker colour and the increase in redness (positive a* value) in muffins with higher CP percentage may have been due to the presence of the carotenoid pigment or to Maillard reactions caused by sugars, proteins, and phenols during baking [40,41]. Greater amounts of CP increased the b* value of the crumb, but no clear trend was observed for the crust. In both the crumb and crust, the b* values were positive, indicating a tendency toward yellow.

Storage induced colour changes for all formulations studied, as deduced from the colour change parameter (ΔE) (Table 5). While colour change in the crust exhibited significant differences among formulations, variations in the crumb were not significant. This fact evidenced that interactions between ingredients and process parameters determined colour, since the crust is the region most exposed to processing and storage conditions.

**Figure 3:**
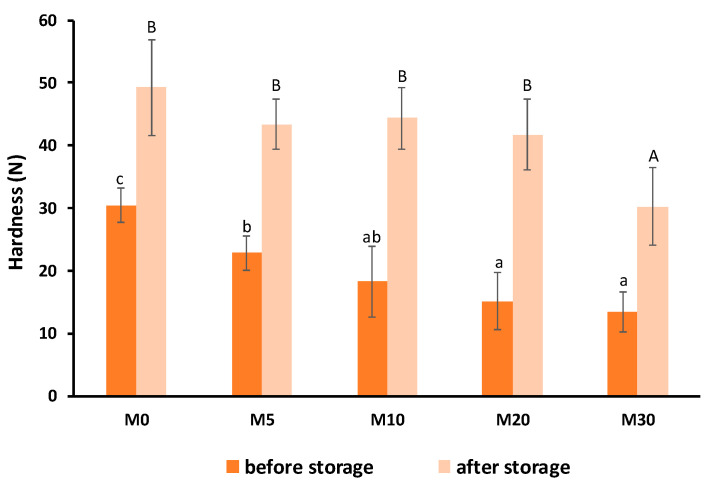
Gluten-free muffin (M) hardness (in N) obtained by replacing rice flour with different percentages of carrot waste powder (0, 5, 10, 20 and 30 indicate the carrot waste powder replacement used in the formulations). ^a,b,c^ or ^A,B^ different letters in the same series indicate significant differences at the 95% confidence level.

**Figure 4:**
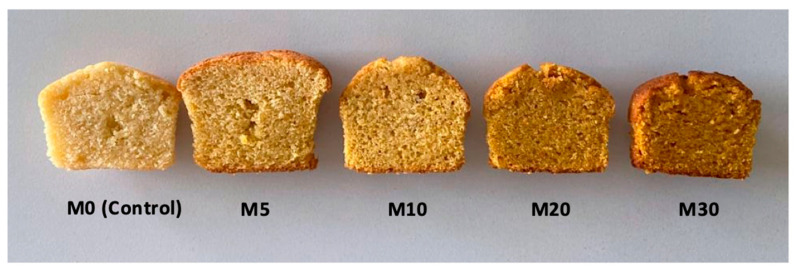
Gluten-free muffin cuts obtained by replacing rice flour with different percentages of carrot waste powder (CP). 0, 5, 10, 20 and 30 indicate the percentage of CP in the flour blend.

Muffin texture was evaluated through hardness, or the highest force required to compress the muffin crumb [16] (Figure 3). The addition of CP in increasing percentages significantly decreased muffin hardness, suggesting a crumblier and softer texture [42]. A softer texture of the muffins could be the consequence of the fibre content of the flours. This trend has also been reported by Bhatt et al. [43] in muffins in which amaranth flour was used to replace black rice flour, for which the softer texture was attributed to the reduced flour porosity and increased density caused by the higher fibre content. Nevertheless, as already mentioned, the pasting properties are also modified by the phenolic constituents added with vegetable powders. Other studies have also shown that the addition of peanut milk powder [44] or potato peel flour [21] decreased muffin and cake hardness; however, opposite results were obtained in other research [16] in which the incorporation of mushroom or carrot powder increased the hardness of rice muffins. The visual appearance of muffin cuts is shown in Figure 4. The images suggested that increasing CP powder induced a more compact structure and volume decrease. This was related to the reduced volume reported by some authors when vegetable powders were added to bakery products [16,17] and would confirm the negative impact of CP on the pasting properties and consequent reduction of dough gas retention ability and leavening. Increased compactness also influences other physical properties, such as colour and texture. As observed, colour variation was the most outstanding difference, with the crust and crumb becoming progressively darker and orangish as the CP proportion increased. The visual appearance aligned with the objective measurement results. Volume and colour are important parameters for bakery products acceptability, and this result should be considered for further product development.

As for the impact of the short cold storage period, hardness significantly increased after one week of storage in all cases. This has been reported in other studies, such as in gluten-free muffins enriched with pecan nut expeller [45] or orange fibre [46]. That increase could be the result of crumb water loss and possible starch retrogradation when cooled, creating an ordered structure with increased hardness [45,47].

**Figure 5:**
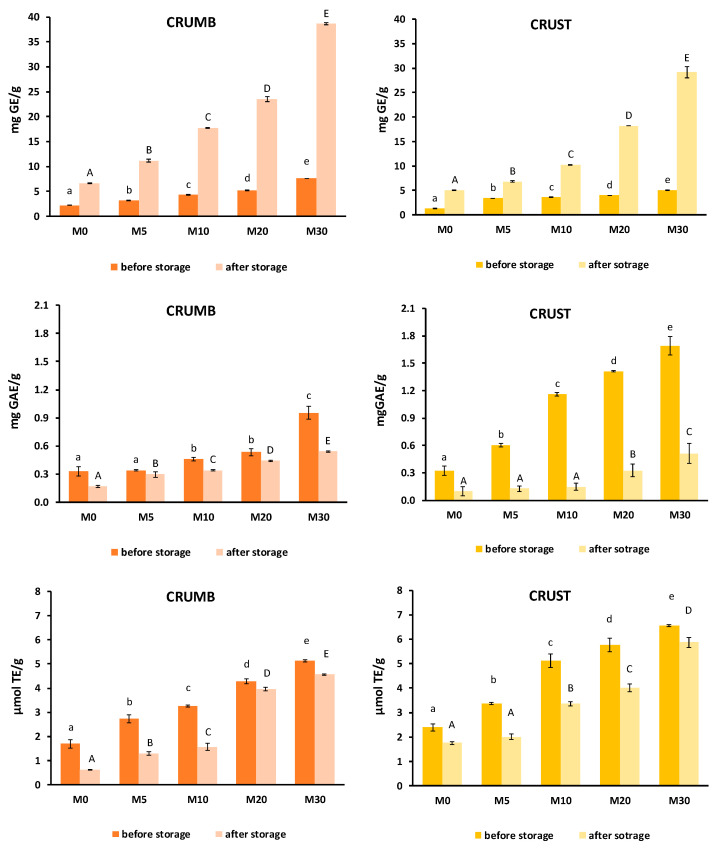
Antioxidant properties of gluten-free muffin crumb and crust obtained by partially replacing rice flour with carrot waste powder (CP) after baking (before storage) and after one week of storage (after storage). 0, 5, 10, 20 and 30 indicate the percentage of CP in the flour blend. (**Upper row**): Reducing sugar content; (**Middle row**): Total phenol content; (**Lower row**): Antioxidant activity by the DPPH method. ^a,b,c,d,e^ or ^A,B,C,D,E^ different letters in the same series indicate significant differences at the 95% confidence level.

The antioxidant properties of the muffins (crumb and crust separately) after baking and after one week of storage are plotted in Figure 5. The reduced sugar content, total phenol content, and antioxidant activity (DPPH) significantly increased with CP addition in both the crumb and crust as compared to the control (rice flour muffin).

A higher amount of CP increased the reducing sugar content from 2.24 ± 0.02 (M0 crumb) or 1.29 ± 0.05 (M0 crust) to 7.61 ± 0.03 (M30 crumb) or 5.08 ± 0.05 mg GE/g (M30 crust), which was mainly attributed to the sugar content of the carrot powder. After one week of storage, values were significantly higher than initial ones, which, according to Singh et al. [48], was explained by the reducing sugars’ capacity to increase when stored at 4 and 8 °C. This trend was also observed in another study with gluten-free breadsticks enriched with white cabbage powder [14].

The increase in total phenol content and antioxidant capacity with CP flour addition was attributed to the phenolic and carotenoid contents of the carrot waste powder [27]. Fortification of bakery products with vegetable powders as functional food ingredients had also been achieved by adding carrot powder and mushroom powder to enriched rice muffins [16], in carrot powder-enriched cookies [33], and in mango peel powder-enriched biscuits [49]. The phenolic content and free radical scavenging (DPPH) results were slightly higher in the crust than in the crumb, which could be attributed to antioxidant compound formation due to Maillard browning during baking [33], as well as to a lower moisture content in the crust, where the compounds present were more concentrated.

Cold storage hindered the antioxidant properties. Phenolic compounds and antioxidant activity (DPPH) were reduced for all formulations and in both muffin crumb and crust. This decrease could be attributed to possible antioxidant degradation reactions [50].

## 4. Conclusions

This study has demonstrated that partially replacing rice flour with IV-range carrot waste powder (CP) in gluten-free formulations can improve the antioxidant and nutritional value of muffins, a popular bakery product consumed worldwide. The use of an upcycled ingredient obtained from food waste, together with this improvement in nutritional properties, confirms that this approach contributes to the circularity of food systems and to more sustainable and healthier diets.

CP addition had a significant impact on the physicochemical properties of the flour blends and muffins obtained, such as pasting, rheological, textural, and colour parameters. The pasting properties generally decreased with CP addition, which could imply a negative impact on the ability of the doughs to retain gas bubbles. This could be significant above a certain flour replacement value and could result in changes in the physical properties of muffins, such as volume or colour. Nevertheless, adding CP significantly improved the antioxidant properties of rice-based products, resulting in enriched and healthier alternatives for those who stick to a gluten-free diet. One week of storage under fridge conditions (4 °C) negatively affected the quality of the products obtained.

In this work, CP obtained from the waste separated in a IV-range carrot line has been revealed as a valuable ingredient to enrich rice flour and elaborate functional gluten-free baked products with improved nutritional properties. However, this is only a first approach to product development. Further research should focus on the sensory evaluation of the products obtained to determine maximum replacement according to consumer acceptability. Additionally, microbiological studies to ensure safety and a complete storage study to determine product stability should also be conducted.

## Figures and Tables

**Table 1:** Moisture content (x_w_), water absorption capacity (WAC), oil absorption capacity (OAC), and hydrophilic/lipophilic index (HLI) of flour blends. CP: carrot powder; 5, 10, 20, 30%: percentage of CP in the flour blend. Mean ± standard deviation of three independent measurements.

Flour Blend	x_w_ (g_w_/100 g)	WAC (mL/g)	OAC (mL/g)	HLI
Rice flour	12.282 ± 0.002 ^c^	2.25 ± 0.03 ^a^	1.57 ± 0.03 ^a^	1.43 ± 0.04 ^a^
CP5%	10.16 ± 0.03 ^a^	2.43 ± 0.05 ^b^	1.66 ± 0.02 ^b^	1.47 ± 0.02 ^a^
CP10%	10.13 ± 0.08 ^a^	2.62 ± 0.02 ^c^	1.64 ± 0.03 ^b^	1.60 ± 0.04 ^b^
CP20%	10.63 ± 0.02 ^b^	3.07 ± 0.12 ^d^	1.66 ± 0.05 ^b^	1.85 ± 0.12 ^c^
CP30%	10.5 ± 0.4 ^ab^	3.56 ± 0.12 ^e^	1.680 ± 0.010 ^b^	2.12 ± 0.06 ^d^

^a,b,c,d,e^ Different superscript letters in the same column indicate statistically significant differences at the 95% confidence level (*p*-value < 0.05).

**Table 2:** Pasting properties of rice flour and powder blends. CP: carrot powder; 5, 10, 20, 30%: percentage of residue powder in the flour blend. PV: peak viscosity; TV: trough viscosity; BD: breakdown; FV: final viscosity; ST: setback; PTp: pasting temperature; Ptime: peak time. Mean ± standard deviation of three independent measurements.

	PV (cP)	TV (cP)	BD (cP)	FV (cP)	ST (cP)	PTp (°C)	Ptime (min)
Rice flour	4498 ± 93 ^e^	3222 ± 66 ^c^	1276 ± 27 ^b^	7353 ± 179 ^c^	4131 ± 112 ^c^	90.55 ± 0.07 ^a^	5.93 ± 0.02 ^a^
CP5%	4033 ± 49 ^d^	3563 ± 5 ^d^	540 ± 54 ^a^	8378 ± 132 ^d^	4816 ± 136 ^d^	89.3 ± 0.7 ^a^	6.4 ± 0.4 ^b^
CP10%	3665 ± 74 ^c^	3130 ± 232 ^c^	535 ± 158 ^a^	7160 ± 35 ^c^	4030 ± 197 ^c^	88.5 ± 0.5 ^a^	6.3 ± 0.5 ^b^
CP20%	3308 ± 21 ^b^	2806 ± 81 ^b^	502 ± 60 ^a^	5803 ± 47 ^b^	2997 ± 127 ^b^	86.8 ± 0.6 ^a^	5.90 ± 0.14 ^a^
CP30%	2782 ± 84 ^a^	2310 ± 56 ^a^	472 ± 28 ^a^	4398 ± 61 ^a^	2089 ± 5 ^a^	85.58 ± 0.04 ^a^	5.89 ± 0.02 ^a^

^a,b,c,d,e^ Different superscript letters in the same column indicate statistically significant differences at the 95% confidence level (*p*-value < 0.05).

**Table 3:** Antioxidant properties of the rice flour and the flours with carrot powder (CP) added. CP: carrot powder; 5, 10, 20, 30%: percentage of residue powder in the flour blend. Mean ± standard deviation of three independent measurements.

Flour Blend	Reducing Sugars (mg GE/g_dm_)	Phenols (mg GAE/g_dm_)	DPPH (µmol TE/g_dm_)
Rice flour	1.257 ± 0.019 ^a^	0.320 ± 0.008 ^a^	4.64 ± 0.05 ^a^
CP5%	8.195 ± 0.018 ^b^	0.677 ± 0.011 ^b^	4.6 ± 0.2 ^a^
CP10%	11.09 ± 0.05 ^c^	1.085 ± 0.002 ^c^	4.54 ± 0.05 ^a^
CP20%	19.14 ± 0.10 ^d^	1.70 ± 0.02 ^d^	5.67 ± 0.05 ^b^
CP30%	21.82 ± 0.12 ^e^	2.30 ± 0.02 ^e^	6.25 ± 0.12 ^c^

^a,b,c,d,e^ Different superscript letters in the same column indicate statistically significant differences at the 95% confidence level (*p*-value < 0.05).

**Table 4:** Water activity (a_w_), water activity after one week of storage (a_w storage_), weight, and volume of gluten-free muffins (M) obtained by partially replacing rice flour with carrot waste powder. 0, 5, 10, 20 and 30 indicate the percentage of carrot waste powder in the flour blend. Mean ± standard deviation of three independent measurements.

	a_w_	a_w storage_	Weight (g)	Volume (mm^3^)
M0	0.895 ± 0.007 ^c^	0.894 ± 0.008 ^c^	42.81 ± 0.07 ^c^	84.3 ± 0.3 ^ab^
M5	0.879 ± 0.004 ^bc^	0.89 ± 0.02 ^bc^	43.0 ± 1.2 ^c^	98 ± 8 ^c^
M10	0.84 ± 0.03 ^ab^	0.866 ± 0.15 ^abc^	41.1 ± 0.7 ^ab^	89 ± 3 ^b^
M20	0.87 ± 0.04 ^bc^	0.86 ± 0.03 ^ab^	42.1 ± 0.3 ^bc^	83 ± 2 ^ab^
M30	0.814 ± 0.007 ^a^	0.846 ± 0.013 ^a^	40.7 ± 0.3 ^a^	81.2 ± 0.4 ^a^

^a,b,c^ Different superscript letters in the same column indicate statistically significant differences at the 95% confidence level (*p*-value < 0.05).

**Table 5:** Colour parameters L*, a*, and b* of gluten-free muffin (M) crumb and crust at time 0 and total colour difference after one week of storage (ΔE). 0, 5, 10, 20 and 30 indicate the percentage of carrot waste powder in the flour blend. Mean ± standard deviation of three independent measurements.

		L*	a*	b*	∆E
CRUMB	M0	63 ± 4 ^d^	−3.1 ± 0.2 ^a^	25.1 ± 0.8 ^a^	8 ± 3 ^a^
	M5	57 ± 3 ^c^	−0.45 ± 0.10 ^b^	32.835 ± 1.006 ^b^	8.7 ± 1.9 ^a^
	M10	58 ± 3 ^c^	1.5 ± 0.5 ^c^	39 ± 4 ^c^	7.0 ± 3 ^a^
	M20	54.2 ± 1.9 ^b^	4.02 ± 0.50 ^d^	44.6 ± 1.5 ^d^	8.3 ± 1.3 ^a^
	M30	50.0 ± 1.6 ^a^	6.1 ± 0.5 ^e^	47.7 ± 1.4 ^e^	9 ± 2 ^a^
CRUST	M0	60 ± 2 ^d^	4.7 ± 1.4 ^a^	34.8 ± 0.4 ^ab^	7.9 ± 1.2 ^a^
	M5	46 ± 3 ^b^	15 ± 4 ^b^	32 ± 4 ^a^	12.1 ± 6 ^ab^
	M10	47.7 ± 0.8 ^bc^	16.2 ± 1.6 ^b^	35.8 ± 0.8 ^ab^	13 ± 2 ^ab^
	M20	50.52 ± 1.17 ^c^	15.5 ± 1.9 ^b^	39 ± 2 ^b^	16 ± 5 ^b^
	M30	40.5 ± 1.5 ^a^	16.5 ± 1.4 ^b^	32.5 ± 0.5 ^a^	11.8 ± 1.3 ^ab^

^a,b,c,d,e^ Different superscript letters in the same column indicate statistically significant differences at the 95% confidence level (*p*-value < 0.05).

## Data Availability

The original contributions presented in the study are included in the article, further inquiries can be directed to the corresponding author.
